# “Motherhood forced me to cope with my disability”: identity intersection among mothers with physical disabilities

**DOI:** 10.3389/fpsyg.2024.1430412

**Published:** 2024-09-25

**Authors:** Carmit-Noa Shpigelman, Limor Karlinski Argi

**Affiliations:** ^1^Department of Community Mental Health, University of Haifa, Haifa, Israel; ^2^The Center for Mental Health Research, Practice, and Policy, University of Haifa, Haifa, Israel

**Keywords:** motherhood, physical disability, intersection, identity, wellbeing

## Abstract

**Background:**

Although motherhood plays a meaningful role in the formation of a woman’s identity, most studies have focused on the process of identity transformation in the transition to motherhood among non-disabled women; less is known about this process among women with physical disabilities who become mothers.

**Objective:**

The present study aimed to understand and describe the subjective experiences of Israeli women with lifelong physical disabilities in their motherhood journey from the perspective of the intersection of their motherhood and disability identities, and from the disability studies approach.

**Methods:**

Semi-structured interviews were conducted with 20 Israeli mothers with visible lifelong physical disabilities who live in the community and raise their children.

**Results:**

Three themes emerged from the interviews: (1) the decision to become a mother: Coping with the disability identity for the first time; (2) The FIRST 3 years: Depending on others as limiting their motherhood identity; (3) after age three: Balancing the motherhood and disability identities.

**Conclusion:**

The transition to motherhood led to identity transformation among women with physical disabilities. Becoming a mother increased the tension between dependence and independence in the context of disability, which also influenced the intersection of their motherhood and disability identities and their wellbeing. Practitioners should provide emotional support to mothers with disabilities and help them embrace the positive aspects of each identity and strike a balance between them.

## 1 Introduction

Motherhood is universally perceived as a key component of women’s identity. An abundance of research exists on the transition to motherhood and on how this process affects women’s identity (Greenberg et al., 2016; Laney et al., 2015; Mercer, 2004; Prinds et al., 2014). The literature on motherhood mainly refers to women’s identity development in the context of relationships and to how to engage in a new type of relationship characterized by dependency (Gilligan, 1982; Laney et al., 2015). In the motherhood literature, dependency mainly refers to the mother–child relationship, while mothers with lifelong disabilities may experience it in a different manner. In the disability studies literature, dependency is discussed in a wider and critical perspective, in relation to the person’s interaction with others (Fine and Glendinning, 2005). In this sense, less is known about the experiences of mothers with lifelong physical disabilities from the intersectionality and disability studies approaches. Understanding the identity transformation process among mothers with physical disabilities is needed to promote the development of interventions that will support their successful transition to motherhood and leverage their wellbeing.

Historically, eugenic, medical, and individualistic approaches to disability, common at the end of the nineteenth and well into the twentieth century, expressed fear of having people with disabilities reproduce and act as parents (Dorfman, 2015; Pfeiffer, 2006). The reproductive roles traditionally reserved for women excluded women with disabilities. Women with disabilities were perceived as incapable of becoming mothers and raising children (Begley et al., 2009; McConnell and Phelan, 2022). Besides these stigmatic attitudes, sterilization policies prevented women with disabilities from exercising their parenting rights (Powell, 2021).

Over the past decades, this paradigm has shifted from over-medicalization to equality approaches. The latter approaches include the social model of disability, which focuses on stigmatizing attitudes toward people with disabilities, and views disability as a phenomenon dependent on wider social contexts rather than solely on the medical-pathological aspects of the individual (Oliver, 2013). Another recent approach is the human rights approach (Shakespeare, 2013), which calls not only to enforce anti-discriminatory laws and regulations, but also to promote accommodations and an inclusive environment to ensure full and meaningful participation of people with disabilities in all life domains. In accordance with this approach, the Convention on the Rights of Persons with Disabilities (CRPD), specifically Article 23, is the first international document calling to promote equal rights for persons with disabilities in the areas of parenthood and family (United Nations General Assembly, 2006).

Despite the rise of the social and human rights models of disability, pregnant women with disabilities are still subjected to strong pressure from their families and medical professionals to have an abortion and to avoid another pregnancy. Mothers with disabilities still report experiencing a lack of accessible information during their pregnancy and after delivery, affecting their wellbeing (Revell, 2019; Schooley, 2013; Signore et al., 2011).

In addition, new parents report discriminatory policies regarding child removal (Powell, 2021). The attitudes of welfare and medical services toward mothers with disabilities, in particular, are affected by stigmas. For example, Mason (2012) described the experiences of 25 women with a visible physical disability, such as blindness, cerebral palsy (CP), and various nervous disorders from the moment they decided to become mothers until their children became adolescents. These mothers reported that many doctors doubted their ability to be good mothers and persisted in viewing them as “sick” women who needed to get well rather than women with ambitions for their own and their children’s future. The doctors tended to recommend an abortion, some out of their fear of the unknown and some out of a more specific fear that the disability would pass on to the children, even when the likelihood for that was low. These attitudes naturally weakened these women’s self-efficacy as mothers.

Mothers with physical disabilities also report avoiding tasking their children with household chores out of fear of being perceived as exploiting them due to their disabilities. They feel constantly scrutinized by the community and school, as well as by family and friends, and feel obligated to prove their parental efficacy at any given moment (Prilleltensky, 2003). Consequently, they often avoid seeking help out of fear their request would be interpreted as indicating lack of competence and their children would be removed from their custody.

Following the affirmative model of disability, which presents a non-tragic view of disability and focuses on disability as positive individual and collective social identities (Swain and French, 2000), some studies have highlighted the positive aspects of motherhood for women with disabilities. It was found, for example, that women with disabilities experienced their pregnancy and childrearing as empowering processes contributing to their self-efficacy and overall wellbeing (Shpigelman, 2015; Shpigelman and Bar, 2023; Farber, 2000; Walsh-Gallagher et al., 2012).

In general, identity in the context of disability has been examined in relation to people with disabilities as a minority group. People with disabilities are confronted with the complex task of developing their identity within the context of the majority, non-disabled group (Shmulsky et al., 2021). This may increase their distress (Seng et al., 2012). The literature on the identity of people with disabilities (Darling, 2019; Dirth and Branscombe, 2018; Forber-Pratt et al., 2022) has often used the term “disability identity,” defined by Dunn and Burcaw (2013) as a positive sense of self, of being part of the disability community. However, less is known about the intersectionality of disability identity with other identities, and in particular, the motherhood identity.

While the transition to motherhood has been historically romanticized and celebrated as a woman’s significant lifetime achievement and a meaningful phase in the woman’s identity development (Forsythe, 2021), disability has been perceived as an undeserved and stigmatized label, which also has a negative impact on the individual’s self-concept (Babik and Gardner, 2021; Nguyen et al., 2020; Trani et al., 2020). Women with disabilities experience double discrimination on the grounds of gender and disability, especially in reproductive healthcare (Casebolt, 2020). Still, these two categories of motherhood and disability have received scant attention in the context of identity development. The present study seeks to deepen our understanding of the identity transformation of women with lifelong physical disabilities as they become mothers. Specifically, it examines the subjective experiences of Israeli women in their motherhood journey from the perspective of the intersection of their motherhood and disability identities and through the lens of disability studies, specifically the International Classification of Functioning, Disability and Health (ICF; World Health Organization, 2001), as described below.

The intersectionality approach refers to all the potential identities that the individual holds, the interactions of these identities with one another, and the systemic contexts within which they are situated (Crenshaw, 1991). Note that although intersectionality has generally focused on oppressed and stigmatized identities such as disability, gender, and ethnicity, some members of disadvantaged groups may also hold privileged identities (Darling, 2019). In this sense, the motherhood identity is perceived as a positive one among the general population. Furthermore, the ICF (World Health Organization, 2001) refers to the individual’s health status from a biological, individual, and social perspective, and also emphasizes the importance of personal (e.g., gender, age, ethnicity, impairment, and personal traits) and contextual factors (e.g., the environment—family, education, employment, services available in the community, and culture). Personal and contextual factors should be taken into consideration in the identity transformation process among mothers with physical disabilities.

The present study focuses on the experiences of Israeli mothers with physical disabilities. In Israel, about 1.7 million people have a disability (18% of the general population). According to the National Insurance Institute of Israel, 32% of people who receive disability benefits are parents with physical disabilities (38,449 parents). Israeli society has sanctified reproduction as a supreme value during the nation-building period, and remains highly pronatalist. Although the State of Israel ratified the CRPD in September 2012 and the state encourages and promotes marriage and childbirth, there are no official policies, services or budgets for parents with disabilities (Dorfman, 2015; Rothler, 2017; Rothler and Efrati, 2021).

## 2 Materials and methods

A qualitative descriptive phenomenological approach was applied to gain a deeper understanding of the lived experiences of the motherhood journey of women with lifelong physical disabilities from the perspective of the intersection of their motherhood and disability identities (Giorgi, 2009; Starks and Trinidad, 2007).

### 2.1 Participants

The sample included 20 Jewish-Israeli women who had met the following criteria: (1) an adult woman (over the age of 18) who is also a Hebrew native speaker, (2) with a diagnosis of lifelong visible physical or motor disability from birth or up to age 20, (3) who has children, and (4) lives in the community (no guardian). The participants were located by professionals working with people with disabilities in the community and through ads in social media groups for adults with physical disabilities. Ethically, the professionals could not disclose the diagnosis, but they orally reported on the eligibility of the women to participate in the study based on the inclusion criteria. Table 1 presents the participants’ demographic data. As indicated in Table 1, the participants’ ages ranged from 29 to 69 years (*M* = 43.95, *SD* = 9.34). They had one to four children; about half (9) had two. Most (11 of 20) were married, six were divorced, and three were single mothers by choice. Note that although some of the participants had more than one child, in the interviews they referred to their transition to motherhood with their first child.

**Table 1:** Participant demographics (*n* = 20).

Pseudonym	Age	Disability	Marital status	No. of children	Ages of children
Abigail	29	CP	Married	1	1.5
Amelia	42	CP	Single mother by choice	1	2
Ayala	35	MS	Married	2	1, 4
Carol	46	CMT	Divorced	2	12.5, 13.5
Dalia	47	Nervous system paralysis	Married	4	19, 20, 24, 27
Edith	40	CP	Single mother by choice	1	6
Elena	51	CP	Married	2	17.5, 20
Iris	30	Static paraplegia	Married	1	7 months
Jane	36	Genetic disease	Married	2	1.4, 3.5
Lily	37	CP	Married	2	5, 7
Luna	50	Leg amputation	Divorced	2	18, 22
Natalia	69	Polio	Divorced	4	36, 27, 44, 44
Nora	47	CP	Divorced	3	16, 22, 23
Olivia	53	Nervous system paralysis	Divorced	1	21
Ruby	44	Leg amputation	Married	3	6, 12, 16
Sarah	49	CP	Single mother by choice	2	11, 11
Shirley	53	Leg amputation	Divorced	1	31
Sophia	45	Right hemiplegia	Married	2	11.5, 15
Tamara	36	Peripheral muscular dystrophy	Married	2	1, 3.5
Zoe	39	CP	Married	3	6, 10.5, 13

*CP, Cerebral palsy; MS, Multiple sclerosis; CMT, Charcot–Marie tooth.

### 2.2 Data collection

Semi-structured interviews were conducted with the participants for about 90 min each. The interview guide developed for this project included several questions related to the participants’ motherhood journey in the past and present, particularly about the women’s self-perception in the context of their mothering role and disability. For example, “How do you perceive and define your motherhood?”; “Do you see your disability as part of your identity or not? (please explain)”; “Have you changed the way you perceive and refer to your disability over the years, and in particular after becoming a mother?”; and “What have you learned about yourself since becoming a mother?” Due to the COVID-19 pandemic, all interviews were conducted via Zoom. The interviews were audiotaped and transcribed verbatim.

### 2.3 Data analysis

Inductive thematic analysis was undertaken to gain an in-depth understanding of the participants’ lived experiences. First, one of the researchers coded the transcripts to identify meaningful units of content. In the second phase, she sorted the codes into potential themes. Next, the other researcher audited the analysis to increase its trustworthiness. The fourth phase involved reviewing and refining the identified themes by the two researchers. The codes associated with each theme were reread to verify that they formed a coherent pattern, and initial themes were revised as needed. Finally, the entire dataset was reread to refine the themes and to determine the relationships between the themes (Braun and Clarke, 2006; Patton, 2015).

To promote the credibility and transferability of the findings, we provided a thick description of the phenomenon under study by quoting excerpts from the participants’ responses (Shenton, 2004). We also applied rigorous data collection to ensure the study’s dependability. As for the data confirmability, we implemented an audit trail by documenting the coding schema and used investigator triangulation to ensure the data analysis was not biased (Creswell and Miller, 2000). In terms of reflexivity, one of the authors is a mother with a lifelong physical disability while the other does not have a disability. A research team consisting of disabled and non-disabled investigators who are also mothers was engaged in discussions on the data analysis, contributing to the authors’ reflexivity regarding their own positions and beliefs about the phenomenon, and reducing the potential of bias in data coding and analysis.

### 2.4 Ethics statement

The research protocol was approved by the Ethics Committee of the Faculty of Social Welfare and Health Sciences at the authors’ university (blinded for review). The women volunteered for this study after providing their oral informed consent at the beginning of the online interview. The consent form included contact details of the research team, especially the second investigator, who has certified training in parent counseling from a well-known Israeli institute. The consent also included phone numbers of Israeli NGOs that provide emotional support. The participants could contact the investigators in case of psychological discomfort as a result of the interview. The participants’ statements presented below are pseudonymized, and all personal identifiers have been omitted to ensure confidentiality.

## 3 Results

The thematic analysis of the interview transcripts revealed three themes related to the mothers’ subjective experience from the intersectional perspective. The themes describe the identity transformation process over different stages of the motherhood journey: the decision to become a mother, the first 3 years after the delivery, and after age three.

### 3.1 The decision to become a mother: coping with the disability identity for the first time

The majority (16 out of 20) reported that in the pre-parenthood period, they felt that their disability was not a significant part of their identity, if at all. They functioned independently, lived in their own apartment in the community, acquired higher education, and worked. They never felt defined by their disability. Throughout their lives, they felt a strong need to be like everyone else, to the point of sometimes giving up on accommodations offered by the environment. Abigail described it as follows: “I did everything like everyone else in my life. I was in junior high like everyone, in high school like everyone.” Similarly, Zoe said: “My disability prevented me from nothing. For starters, I studied in a mainstream high school from fifth grade, socialized with ‘normal’ kids, went to parties, to a youth movement […] went on to higher education.” Jane emphasized that she had given up on the accommodations to which she was entitled so as not to feel different: “They wanted to exempt me from physical education, and I forced them to test me.” It seems that these women did not want to be perceived and treated as disabled, so they refused to belong to the disability community or obtain accommodations. Nora described it: “In retrospect, the disability played a very big role in my life. The disability actually managed my life, but I did not realize that then [when I was a child]. I felt I had to be like everyone else [non-disabled].” For some, the desire to be like everyone was also expressed in avoiding social contact with others with disabilities: “I did not see the disability, and not only did not I see it, I also refused to spend time with people with a disability” (Elena).

Moreover, the participants had a lifelong sense that they had to prove to themselves and others that they were equal. Shirley said: “I proved to myself, to the world, to my family and friends—I was always in a ‘proving’ state of mind. With all the difficulties and injuries, the operations and hospitalizations, I was never easy on myself, not one bit.” Sarah expressed a similar sentiment: “This situation [the disability] really forces you all the time to prove that you are a worthy and good-enough person.” As opposed to the human rights approach, it seems that equality was perceived by these women as overcoming their disabilities and the associated difficulties. They focused on blurring their impairments in order to get close and feel they belonged to the non-disabled group. Some of them described that the pressure to overcome their disability and act like non-disabled women came first from their families, especially their parents. Luna described it: “My parents did not make any concessions for me. I mean, not at school, not at work; I had to get married and have children. They had a checklist, and they really treated me like any other child in the family.”

As the women grew, they viewed motherhood as a natural process expected of them just as it was expected from non-disabled women. Zoe described it as follows:

All my life, I was a person who … who really did everything. […] and I told myself I would do anything to become a parent […] on the whole, my disability was never a part [of my identity] I don’t want it to sound like I deny the disability. I really don’t. But it’s like I feel it’s not something that dictated my life course.

These women became much more keenly aware of their disability, however, when they decided to have children. They began to think and refer to their disability, still in a negative manner, when they decided to become a mother. The great majority (18 out of 20) shared that deciding to be a mother was accompanied by various fears related to their disability, particularly with regard to caring for the infant by a mother or two partners with disability. For instance, Carol feared the responsibility involved, particularly in situations where the infant would be in danger:

We didn’t get married, by the way, but my partner wanted, even before I felt ready. It made me nervous, I really felt I was still a little girl {laughing}, but I wasn’t […] how was I to take care of babies, and hold them, and lift them […]. And I was afraid […] and he said he’d help me […]. I was always scared by the responsibility, that God forbid something would happen and I won’t be able to help my children. […] Say the child is choking and I can’t put my fingers into his mouth and pull out whatever is there. Say a child runs into the street, and I can’t chase him.

Some of the participants shared a self-stigma expressed in fear of passing the disability on to the child, particularly when it was genetic. For example, Tamara stated that at a young age, and had never even considered having children: “Certainly. This was certainly a question. Until a relatively very advanced age, I did not even … consider having children. I always said I will not have children because I do not want them to inherit my disease.”

Moreover, even when the disability was not hereditary in any way, some of the women feared their child would be disabled, for any reason whatsoever. Amelia, for example, confessed by having “a hysterical fear of having a child with a disability, because how can I contain a child with a disability with my own disability?”

Early on, after deciding to have children, their physical disability became more present. For about half of women, the pregnancy was accompanied by a temporary or permanent exacerbation of their physical disability, which had a negative effect on their wellbeing. Lily highlighted the degree to which her disability intensified during her pregnancy—both physically and emotionally:

Look, first of all, physically, it’s very hard being pregnant and being with a physical disability, and as the pregnancy progresses, the difficulty intensifies. To the point, I could hardly walk […] could hardly wear socks at some point … could hardly bend over.

Some of them experienced the pregnancy as a positive process, although it was accompanied by a deterioration in their health. For the first time, they loved their looks, their body, as Dalia described: “I think that was a time when I loved myself the most. I saw it [pregnancy] as something very beautiful; I was very proud of my body; I loved it very much.”

### 3.2 The first 3 years after: depending on others as limiting their motherhood identity

As mentioned in the previous theme, by the time of delivery, most women experienced their disability as part of their lives. They were aware of it, but did not live it. Their disability was not integral to their identity in terms of their self-perception. However, more than half (11 out of 20) reported that they made extensive logistical preparations for the day after in terms, such as purchasing assistive technologies that could help them treat the infant independently, hiring a caregiver, and gathering information about government services or NGOs that could help them. In short, they looked for accommodations that would enable them to keep functioning independently. For example, Tamara said she did not want to be completely dependent on another, so she found out everything she could about devices that could help her as a mother with disability:

I wanted to be independent. I mean, you know, I clearly need help with some things, but yeah, to manage alone, at least for several hours a day or at least in some of the activities […] I would not have had it any other way […], but then there were all kinds of fears around how I would manage, like, to cope with all kinds of physical things that I’d have to do because I was not willing to be completely dependent on anyone who would help me be a mother […] as a person who’s very rational and very aware of her limitations and ability, I conducted a very significant preparatory work before delivery […] so I went and researched about all kinds of equipment that could help me and thought things through down to the last details […] if I dress up the baby, how am I going to close the leotard, because I can’t close”.

Thus, although Tamara was technically well-prepared, the fear of depending on others exacted a high emotional price from her.

After the birth, the majority (12 out of 20) felt their motherhood enabled them to prove that they were just like anyone else. On the one hand, the delivery and their new parental status blurred the disability part in their identity. For example, Ruby felt empowered by her pregnancy, delivery, and motherhood:

Gee, I can be pregnant just like everybody else. I can really have a baby like everyone, even if it is a cesarian. I can raise the kids afterward—hugely empowering […] a boost to my self-esteem. Because it really is an achievement.

On the other hand, despite this positive experience and the practical preparations made in advance, in the first 3 years of parenthood, the intensive care of the infant, together with the dependency on others, made many of the participants feel helpless and emotionally distressed as a result. Being dependent on others made their functional difficulties much more apparent, and made them feel frustrated for not being able to be the mothers they wanted to be. Their disability became central to their identity as their motherhood identity developed. Edith described it as follows: “Then suddenly [after the delivery] it was like the disability hit me like a boomerang. Suddenly, I felt that there were so many things I could not do on my own. This was my first crisis in parenthood.” Edith went on to describe the extra burden she experienced because she had to take care of herself and her baby girl: “It’s about caring for myself in this state of dependency and lack of control, while also caring for her […] ‘too much’ for me.”

Amelia also said that prior to becoming pregnant, she had perceived herself as an independent woman. After the delivery, however, she felt she was losing her independence completely, which in turn negatively influenced her wellbeing:

I really didn’t know where to bury myself […]. Total helplessness […] I was independent only a minute ago […] it kind of contrasted with the fact that before that, it [the disability] hardly had any room. I mean it was very repressed […] being a parent made me a lot more … I now had to constantly cope. I mean, I couldn’t get away from it, I couldn’t ignore it, I couldn’t forget. […] it’s constantly in front of me.

Most of the participants (12 of 20) also described how the people helping them with the baby, including close relatives and paid caregivers, questioned their parental competence, which only exacerbated their emotional distress. Amelia described this as follows:

Now, I want certain things, and my mom or whoever it is who’s helping me take care of her at that moment wants it otherwise, or things otherwise. Then suddenly, I become [disabled] because I’m her mom, but actually, I don’t have a real say. I need to ask everything from somebody else … There were times I simply said, alright, enough, can’t do it any longer. […] I didn’t know what to do […] I didn’t live in my mother’s house, didn’t ask her each time before going to places […]. And suddenly I have to tell her about every tiny little thing, and not only that, but also obtain her approval […] all of a sudden, your opinion is just one opinion. Some people don’t think like me, and they are also the ones who would eventually decide.

Others expressed concern with the fact that the paid caregiver was “taking over.” In some cases, it was only the mothers’ fear, as Elena described: “I almost did not let her touch my baby. I mean, except for bathing him, because it was really hard for me at first. I was so afraid that she would take the mothering role away from me.” In other cases, the mothers described a situation in which the baby became attached to the caregiver:

The moment she left the house, my daughter would grab her leg, cry, and not want to let her go. I also noticed that my daughter became alienated from me in every possible way. She didn't want kisses from me, didn't want to hug me, and didn't want me to come near her (Abigail).

### 3.3 After age three: balancing the motherhood and disability identities

As the children grew and became independent, their mothers managed to strike a balance between their identities, such that their disability remained present next to a motherhood identity which became more dominant. The women’s concept of an ideal mother changed with time. Lily described how she came to realize that her limited functioning did not affect her daughters:

It’s simply more difficult. So we do more of other things, and I don’t think my daughters have missed anything in their childhood experience because we do travel […]. I had to work on myself to get there. And it took me time to realize, to do the process with myself and realize, OK, some things you can and some things you can’t. It did help me a lot to think that they were no different from any other kids in their environment […].

Iris also emphasized how she came to realize over the years that she could give her child things her paid caregiver would never be able to give:

Look, to be a good mom, you don’t really need all the … it’s not about the physical thing. […] in terms of warmth and love and in terms of education and intellect—the caregiver, after all, cannot sit down and read the book and things like that, which I, for example, very much love doing. So it’s like in these moments […] she’s with me, she [the caregiver] can never take it away from me. […] the technical stuff, she can do that […].

It seems that these women found creative ways of giving to their children as mothers, including accessible activities:

Today, I see it [motherhood] as the best title; […] the most important title. I have learned that I should try, even if something seems to me threatening and scary at first, because of my disability. I always try […] to find a solution, always try to think, how do I do it together with my disability? It [my disability] really motivated me to look for solutions (Zoe).

Regarding the identity balance, the majority (15 out of 20) considered the motherhood role as an opportunity to construct a more positive disability identity. They felt parenting posed new challenges for them, an opportunity for growth and development in relation to their disability, and consequently learned to live with and accept it. Nora described it as follows: “I tried to cope in whatever way I could. As a mom, I think I’ve done my best. […] Parenting is an enriching experience […] one way or another, it makes you face your disability, so it’s important.”

Lily emphasized the element of choice in relation to the disability, a choice that also opened up broader possibilities for her daughters to relate to the disability, despite the difficulty she had experienced at first:

Disability affects parenthood, no doubt. But it’s all a question of how you choose to relate to it. I think that with regard to this issue as well, the parents can serve as role models, how I relate to my disability, and this way my daughters also learn to relate to disability themselves.

Apparently, once the children grew, disability was no longer perceived as a major obstacle. At this point, the participants related to their disability in a more balanced way, accepting it on the one hand but feeling that it was shrunk to its previous “size” in their identity. Carol described this mature stage in her identity development as follows:

This place has already become much narrower. Very much so. I hardly ever pay attention to that […] this is who I am and whoever wants me will accept me this way. […] I’m just as good as anyone, in everything. This is something I really felt over the past months, not only rationally—I experienced it […] it's amazing things have come to that; it’s about time.

Ruby also described this: “I got used to the fact that I could do certain things and could not do others. So, I have a disability, but that’s it; there are things I can do and things I cannot.”

Finally, some participants, like Iris, talked about their disability from a human rights perspective:

In the past [pre-parenthood], when I arrived at a parking lot and wanted to park in an accessible space, I would say to the parking lot attendant, “I have a disability card”. I would not tell him, “I’m disabled.” Today, disability is part of who I am. I understand it is part of my identity, and I am able to say, “I’m disabled.” I have rights as a disabled mother, and I enjoy them.

## 4 Discussion

The study aimed to understand and describe the subjective experiences of women with physical disabilities in their motherhood journey from the intersectional and disability studies approaches. The findings indicated a transformative process related to these women’s identities. The identity transformation unfolded over the different stages of the motherhood journey, from the decision to become a mother through the pregnancy and delivery, the first 3 years of the child’s life, and the following years (when the child grew up). These women progressively moved from ignoring or not accepting their disability identity through being aware of the disability alongside the motherhood identity to being able to embrace or accept the two identities and strike a balance between them.

The mothers in the present study experienced the pre-parenthood period and the first 3 years after the delivery in a manner associated with the medical model of disability. The paradigm shift from the medical to the social model and human rights approach (Degener, 2016; Oliver, 2013) did not resonate in the mothers’ experience. They internalized the negative social attitude toward disability and ignored or rejected their disability in order to be accepted in the dominant group of non-disabled women. In fact, throughout their lives, they appear to have done everything strictly according to social norms, including acquiring higher education and employment, and independent living. In Israeli society, characterized by strong familialism and a high birthrate, the choice to have children was part of being like other women, part of adopting the norm. Accordingly, the participants narrowed the place of disability in their identity, to the point of giving up on accommodations offered to them. Some even avoided contact with others with disabilities. This is aligned with Darling (2003) typology of orientation to disability, particularly with the first—normalization.

The delivery and their new status as mothers were a source of great satisfaction—having proven to themselves and the world that they could be just like any other women. Childbirth is described in the literature as a significant life event that arouses mixed feelings among mothers: joy, happiness, and self-fulfillment, on the one hand, and an overwhelming responsibility sometimes accompanied by feelings of shock, crisis, anxiety, and loneliness, on the other hand (Arnold-Baker, 2020; Hennekam et al., 2019). Like non-disabled mothers, the mothers in the present study experienced pregnancy and birth as a meaningful life event (Lawler et al., 2015). They also viewed it as a natural process they wished to experience as women. However, unlike non-disabled mothers, the mothers in the present study had to negotiate this natural process in the context of their disability. For example, while the dependence-independence discourse (Fine and Glendinning, 2005) refers to the mother–child interaction among the general (non-disabled) population, for mothers with disabilities, this discourse mainly relates to the mothers’ interaction with others from the immediate environment, such as family members and paid caregivers. As opposed to non-disabled mothers, the mothers from the present study had to cope with personal challenges, such as deterioration in their physical health due to the pregnancy, and contextual challenges, such as stigmatic beliefs held by their others regarding their parental competency (World Health Organization, 2001).

Furthermore, despite those challenges, it seems that the women in the present study, did not experience their mothering role as a crisis (Arnold-Baker, 2020). On the contrary, they experienced it as a positive process contributing to their self-efficacy and overall wellbeing. They were empowered by the fact that they became mothers despite their disability. This finding demonstrates again the (ability to overcome the) internalization of disability as a stigmatized identity and, as a result, a discredited and unwanted part of the self (Gustavsson and Nyberg, 2015).

The pregnancy—accompanied, for some of the women, by an exacerbation of their physical disability, making them dependent for the first time on others’ help—highlighted their disability part in their identity. As found in other studies (Shpigelman, 2015; Heideveld-Gerritsen et al., 2021; Prilleltensky, 2003), the mothers in the present study prepared in advance in order to make childcare more accessible, such as purchasing certain devices and assistive technologies or employing a caregiver. Following the social model of disability (Oliver, 2013), the women accommodated the environment to their needs. However, despite these preparations, they confronted a harsh reality after birth, as they required more help than expected. This made them feel helpless and exacerbated their distress. They appeared to accept the common social view of functional independence as an indicator of success, particularly in parenting. The dependence-independence discourse intensified after the delivery and during the first 3 years of the child’s life. The negative view of disabled people’s dependence persists, despite being inconsistent with the social model of disability that calls for abandoning the dependence-independence dichotomy and adopting the notion of interdependence (Fine and Glendinning, 2005).

This high dependency on others after birth disrupted the balance between the participants’ motherhood and disability identities, such as the latter expanded at the expense of the former. This finding can be explained by Crenshaw’s intersectionality theory (1991) and the understanding that the disability identity can also be intersected with privileged identities such as motherhood (Cole, 2009). The women in the present study perceived their motherhood identity as empowering by enabling them to belong to a non-disabled society. In contrast, their disability identity was perceived as highlighting their social difference.

Note that the women’s motherhood identity was also limited by the stigmatic reactions of their family members, who provided physical assistance but also took control of the child and did not allow the women to make decisions regarding them. The women perceived it as overprotection. They felt that this situation where their family cared for their children detracted from their motherhood experience, resulting in increased frustration and reduced self-efficacy and self-esteem as parents. This finding further supports previous studies indicating that family overprotection affects the ability of persons with disabilities to make their own decisions in various life domains (Shpigelman and Bar, 2023; Nosek et al., 2003; Sanders, 2006).

Gradually, however, as the child grew and as the women became more adjusted to their motherhood role, they learned to accept their disability as part of their identity. This was enabled by a changed attitude to the concept of dependency. With time, they learned how to value their emotional giving to the child, even if another person was in charge of the child’s physical care. They now perceive disability as a given, challenging their parenting but not disrupting it—an opportunity for personal growth. Most of them came to see motherhood as an opportunity to construct a more positive disability identity. This finding demonstrates the affirmative model of disability, which views disability from an empowering perspective. It does not deny that there can be negative experiences resulting from living with an impairment, but this is not all that impairment is about (Cameron, 2015; French and Swain, 2008).

While on the macro level, the discourse related to people with disabilities is based on human rights, on the micro level, the mothers’ identity has been mainly constructed by their life experience, including personal factors, such as health status, and contextual factors, such as stigmatic beliefs and reactions. In the pre-parenthood period and early stages of motherhood, the mothers perceived and referred to their disability from a medical perspective while viewing it as a normative and desirable identity. Over the years, especially as the children grew and became independent, the mothers learned to embrace their disability and balance their different identities. Overall, the present study’s findings support the argument that the disability identity should not be viewed as a dichotomy of disabled vs. disabled. Rather, it should be understood as multiple, complex, and dynamic (Gustavsson and Nyberg, 2015; Shakespeare, 1996). Accordingly, women with disabilities can experience the interplay of their disability and motherhood identities as both stigmatic and positive.

### 4.1 Limitations and future directions

This study contributes to our understanding of the motherhood journey of women with physical disabilities from the intersectional and disability studies perspectives. Nevertheless, it has several limitations that need to be addressed in future studies. First, due to the lack of an official database and policy in the area of parenthood among Israeli adults with disabilities, it was difficult to locate candidates for the current study, so the mothers were recruited using the snowball technique, which limits the participants’ heterogeneity and the ability to generalize from the findings (Parker et al., 2019). Indeed, most of the participants were higher-middle-class educated professionals. Second, the women’s age range was rather wide (29–70), affecting the older women’s ability to recall and retrospectively report their perceptions and experiences. In a future study, we recommend sampling a homogenous group of mothers with younger children (up to the age of 18). It is also recommended to conduct a longitudinal study examining the identity transformation process as the children develop. In addition, we recommend examining the identity issue while comparing women with disabilities with disabled vs. non-disabled partners.

### 4.2 Implications for practice

The findings highlight the need to support women with physical disabilities in emotional and identity processes related to pregnancy, delivery, and childrearing, particularly in the first few years, to enable them to strike a balance between their motherhood and disability identities. The construction of a disability identity should ideally begin before parenthood, in order to enable these women to enter parenthood from a more emotionally stable position. Finally, welfare and health service providers should instruct family members and others assisting mothers with disabilities on how to support their needs in a respectful approach and from a human rights perspective, while enabling the mothers to make childcare decisions on their own.

## Data Availability

The raw data supporting the conclusions of this article will be made available by the authors, without undue reservation.

## References

[ref1] Arnold-Baker C. (2020). The existential crisis of motherhood. Cham: Springer Nature.

[ref4] Babik I. Gardner E. S. (2021). Factors affecting the perception of disability: a developmental perspective. Front. Psychol. 12:702166. doi: 10.3389/fpsyg.2021.702166, PMID: 34234730 PMC8255380

[ref5] Begley C. Higgins A. Lalor J. Sheerin F. Alexander J. Nicholl H.. (2009). Women with disabilities: Barriers and facilitators to accessing services during pregnancy, childbirth and early motherhood. Dublin: National Disability Authority.

[ref6] Braun V. Clarke V. (2006). Using thematic analysis in psychology. Qual. Res. Psychol. 3, 77–101. doi: 10.1191/1478088706qp063oa

[ref7] Cameron C. (2015). Turning experience into theory: the affirmation model as a tool for critical praxis. Soc. Work Soc. Sci. Rev. 17, 108–121. doi: 10.1921/swssr.v17i3.802

[ref8] Casebolt M. T. (2020). Barriers to reproductive health services for women with disability in low-and middle-income countries: a review of the literature. Sex. Reprod. Healthc. 24:100485. doi: 10.1016/j.srhc.2020.100485, PMID: 32036280

[ref9] Cole E. R. (2009). Intersectionality and research in psychology. Am. Psychol. 64, 170–180. doi: 10.1037/a0014564 19348518

[ref10] Crenshaw K. (1991). Mapping the margins: intersectionality, identity politics, and violence against women of color. Stanford Law Rev. 43, 1241–1299. doi: 10.2307/1229039

[ref11] Creswell J. W. Miller D. L. (2000). Determining validity in qualitative inquiry. Theory Pract. 39, 124–130. doi: 10.1207/s15430421tip3903_2

[ref12] Darling R. B. (2003). Toward a model of changing disability identities: a proposed typology and research agenda. Disability Soc. 18, 881–895. doi: 10.1080/0968759032000127308

[ref13] Darling R. B. (2019). Disability and identity: Negotiating self in a changing society. Boulder, CO: Lynne Rienner.

[ref14] Degener T. (2016). “A human rights model of disability” in Handbook of disability law and human rights. eds. Blanck P. D. Flynn E. (London: Routledge), 31–49.

[ref15] Dirth T. P. Branscombe N. R. (2018). The social identity approach to disability: bridging disability studies and psychological science. Psychol. Bull. 144, 1300–1324. doi: 10.1037/bul0000156, PMID: 29999335

[ref16] Dorfman D. (2015). The inaccessible road to motherhood-the tragic consequence of not having reproductive policies for Israelis with disabilities. Columbia J. Gend. Law 30, 49–83. doi: 10.7916/cjgl.v30i1.2726

[ref17] Dunn D. S. Burcaw S. (2013). Disability identity: exploring narrative accounts of disability. Rehabil. Psychol. 58, 148–157. doi: 10.1037/a0031691, PMID: 23437994

[ref18] Farber R. S. (2000). Mothers with disabilities: in their own voice. Am. J. Occup. Ther. 54, 260–268. doi: 10.5014/ajot.54.3.260, PMID: 10842682

[ref19] Fine M. Glendinning C. (2005). Dependence, independence, or inter-dependence? Revisiting the concepts of ‘care’ and ‘dependency’. Ageing Soc. 25, 601–621. doi: 10.1017/S0144686X05003600

[ref20] Forber-Pratt A. J. Price L. R. Merrin G. J. Hanebutt R. A. Fairclough J. A. (2022). Psychometric properties of the disability identity development scale: confirmatory factor and bifactor analyses. Rehabil. Psychol. 67, 120–127. doi: 10.1037/rep0000445, PMID: 35377698

[ref21] Forsythe S. M. (2021). The perception of motherhood through the smiles and the spit-up (Unpublished MA thesis). Statesboro, GA: Georgia Southern University.

[ref22] French S. Swain J. (2008). “Affirming identity” in On equal terms: Working with disabled people. eds. French S. Swain J. (London: Sage), 65–79.

[ref24] Gilligan C. (1982). In a different voice: Psychological theory and women's development. Cambridge, MA: Harvard University Press.

[ref25] Giorgi A. (2009). The descriptive phenomenological method in psychology: A modified Husserlian approach. Pittsburgh, PA: Duquesne University Press.

[ref26] Greenberg D. N. Clair J. A. Ladge J. (2016). “Identity and the transition to motherhood: navigating existing, temporary, and anticipatory identities” in Research perspectives on work and the transition to motherhood. eds. Spitzmueller C. Matthews R. A. (Cham: Springer International Publishing/Springer Nature), 33–55.

[ref29] Gustavsson A. Nyberg C. (2015). “‘I am different, but I’m like everyone else’: the dynamics of disability identity” in Childhood and disability in the Nordic countries: Being, becoming, belonging. eds. Traustadóttir R. Ytterhus B. Egilson S. Berg B. (London: Palgrave Macmillan UK), 69–84.

[ref30] Heideveld-Gerritsen M. van Vulpen M. Hollander M. Maatman S. O. Ockhuijsen H. van den Hoogen A. (2021). Maternity care experiences of women with physical disabilities: a systematic review. Midwifery 96:102938. doi: 10.1016/j.midw.2021.102938, PMID: 33636618

[ref31] Hennekam S. Syed J. Ali F. Dumazert J. P. (2019). A multilevel perspective of the identity transition to motherhood. Gender Work Org. 26, 915–933. doi: 10.1111/gwao.12334

[ref32] Laney E. K. Hall M. E. L. Anderson T. L. Willingham M. M. (2015). Becoming a mother: the influence of motherhood on women's identity development. Identity 15, 126–145. doi: 10.1080/15283488.2015.1023440

[ref33] Lawler D. Begley C. Lalor J. (2015). (Re) constructing myself: the process of transition to motherhood for women with a disability. J. Adv. Nurs. 71, 1672–1683. doi: 10.1111/jan.12635, PMID: 25691270

[ref35] Mason M. G. (2012). Taking care: Lessons from mothers with disabilities. Lanham, MD: University Press of America.

[ref36] McConnell D. Phelan S. (2022). The devolution of eugenic practices: sexual and reproductive health and oppression of people with intellectual disability. Soc. Sci. Med. 298:114877. doi: 10.1016/j.socscimed.2022.114877 35276622

[ref37] Mercer R. T. (2004). Becoming a mother versus maternal role attainment. J. Nurs. Scholarsh. 36, 226–232. doi: 10.1111/j.1547-5069.2004.04042.x, PMID: 15495491

[ref38] Nguyen W. Ownsworth T. Nicol C. Zimmerman D. (2020). How I see and feel about myself: domain-specific self-concept and self-esteem in autistic adults. Front. Psychol. 11:913. doi: 10.3389/fpsyg.2020.00913, PMID: 32477221 PMC7235351

[ref001] Nosek M. A. Hughes R. B. Swedlund N. Taylor H. B. Swank P. (2003). Self-esteem and women with disabilities. Soc. sci. med. 56, 1737–1747. doi: 10.1016/s0277-9536(02)00169-7, PMID: 12639590

[ref39] Oliver M. (2013). The social model of disability: thirty years on. Disability Soc. 28, 1024–1026. doi: 10.1080/09687599.2013.818773

[ref41] Parker C. Scott S. Geddes A. (2019). Snowball sampling. London: Sage.

[ref42] Patton M. Q. (2015). Qualitative evaluation and research methods. 4th Edn. London: Sage.

[ref43] Pfeiffer D. (2006). “Eugenics and disability discrimination” in Overcoming disabling barriers (London: Routledge), 80–100.

[ref44] Powell R. M. (2021). Achieving justice for disabled parents and their children: an abolitionist approach. Yale JL Femin. 33:37. doi: 10.2139/ssrn.3916265

[ref45] Prilleltensky O. (2003). A ramp to motherhood: the experiences of mothers with physical disabilities. Sex. Disabil. 21, 21–47. doi: 10.1023/A:1023558808891

[ref46] Prinds C. Hvidt N. C. Mogensen O. Buus N. (2014). Making existential meaning in transition to motherhood—a scoping review. Midwifery 30, 733–741. doi: 10.1016/j.midw.2013.06.021, PMID: 23928210

[ref47] Revell M. A. (2019). Challenges of motherhood with physical disabilities. Int. J. Childbirth Educ. 34, 18–22.

[ref48] Rothler R. (2017). Disability rights, reproductive technology, and parenthood: unrealised opportunities. Reprod. Health Matters 25, 104–113. doi: 10.1080/09688080.2017.1330105, PMID: 28784069

[ref49] Rothler R. Efrati Y. (2021). The right to parenthood of people with disabilities: Update and recommendations for action. Commission for Equality of rights for persons with disabilities, the Israeli Ministry of Justice. Available at: https://www.gov.il/he/departments/publications/reports/parenthood_right_report (Accessed November 1, 2022).

[ref50] Sanders K. Y. (2006). Overprotection and lowered expectations of persons with disabilities: the unforeseen consequences. Work 27, 181–188, PMID: 16971765

[ref51] Schooley R. (2013). The "other" mother: mothering with physical disabilities in a disabling society. Footnotes, 6.

[ref52] Seng J. S. Lopez W. D. Sperlich M. Hamama L. Reed Meldrum C. D. (2012). Marginalized identities, discrimination burden, and mental health: empirical exploration of an interpersonal-level approach to modeling intersectionality. Soc. Sci. Med. 75, 2437–2445. doi: 10.1016/j.socscimed.2012.09.023, PMID: 23089613 PMC3962770

[ref53] Shakespeare T. (1996). “Disability, identity and difference” in Exploring the divide. eds. Barnes C. Mercer G. (Leeds: The Disability Press), 94–113.

[ref54] Shakespeare T. (2013). Disability rights and wrongs revisited. London: Routledge.

[ref55] Shenton A. K. (2004). Strategies for ensuring trustworthiness in qualitative research projects. Educ. Inf. 22, 63–75. doi: 10.3233/EFI-2004-22201

[ref56] Shmulsky S. Gobbo K. Donahue A. Klucken F. (2021). Do neurodivergent college students forge a disability identity? A snapshot and implications. J. Postsec. Educ. Disability 34, 53–63.

[ref002] Shpigelman C. N. (2015). How to support the needs of mothers with physical disabilities? Disability and Rehabilitation. 37, 928–935. 10.3109/09638288.2014.948133 25098594

[ref003] Shpigelman C. N. Bar M. (2023). I’m a good mother; I play with her; I love her”: The motherhood experience of women with intellectual disabilities from empowering and intersectional approaches. Disability and Health Journal, 101504., PMID: 37468407 10.1016/j.dhjo.2023.101504

[ref57] Signore C. Spong C. Y. Krotoski D. Shinowara N. L. Blackwell S. C. (2011). Pregnancy in women with physical disabilities. Obstet. Gynecol. 117, 935–947. doi: 10.1097/AOG.0b013e3182118d59 21422868

[ref58] Starks H. Trinidad S. B. (2007). Choose your method: a comparison of phenomenology, discourse analysis, and grounded theory. Qual. Health Res. 17, 1372–1380. doi: 10.1177/1049732307307031, PMID: 18000076

[ref59] Swain J. French S. (2000). Towards an affirmation model of disability. Disability Soc. 15, 569–582. doi: 10.1080/09687590050058189

[ref60] Trani J. F. Moodley J. Anand P. Graham L. Maw M. T. T. (2020). Stigma of persons with disabilities in South Africa: uncovering pathways from discrimination to depression and low self-esteem. Soc. Sci. Med. 265:113449. doi: 10.1016/j.socscimed.2020.113449, PMID: 33183862 PMC7576188

[ref61] United Nations General Assembly. (2006). Convention on the rights of persons with disabilities. Available at: https://www.un.org/development/desa/disabilities/convention-on-the-rights-of-persons-with-disabilities.html (Accessed September 15, 2020).

[ref62] Walsh-Gallagher D. Sinclair M. McConkey R. (2012). The ambiguity of disabled women's experiences of pregnancy, childbirth and motherhood: a phenomenological understanding. Midwifery 28, 156–162. doi: 10.1016/j.midw.2011.01.003, PMID: 21570753

[ref64] World Health Organization. (2001). International classification of functioning, disability and health: ICF. Geneva: World Health Organization

